# Sensing Magnetic Directions in Birds: Radical Pair Processes Involving Cryptochrome

**DOI:** 10.3390/bios4030221

**Published:** 2014-07-24

**Authors:** Roswitha Wiltschko, Wolfgang Wiltschko

**Affiliations:** Fachbereich Biowissenschaften, J.W. Goethe-Universität Frankfurt, Max von Laue Straße 13, D-60438 Frankfurt am Main, Germany; E-Mail: wiltschko@zoology.uni-frankfurt.de

**Keywords:** avian magnetic compass, inclination compass, functional window, Radical Pair Model, cryptochrome, Cry 1a, retina, UV/V cones

## Abstract

Birds can use the geomagnetic field for compass orientation. Behavioral experiments, mostly with migrating passerines, revealed three characteristics of the avian magnetic compass: (1) it works spontaneously only in a narrow functional window around the intensity of the ambient magnetic field, but can adapt to other intensities, (2) it is an “inclination compass”, not based on the polarity of the magnetic field, but the axial course of the field lines, and (3) it requires short-wavelength light from UV to 565 nm Green. The Radical Pair-Model of magnetoreception can explain these properties by proposing spin-chemical processes in photopigments as underlying mechanism. Applying radio frequency fields, a diagnostic tool for radical pair processes, supports an involvement of a radical pair mechanism in avian magnetoreception: added to the geomagnetic field, they disrupted orientation, presumably by interfering with the receptive processes. Cryptochromes have been suggested as receptor molecules. Cry1a is found in the eyes of birds, where it is located at the membranes of the disks in the outer segments of the UV-cones in chickens and robins. Immuno-histochemical studies show that it is activated by the wavelengths of light that allow magnetic compass orientation in birds.

## 1. Introduction

In the 1960s, it was first discovered that animals can sense the direction of the geomagnetic field and use it as a compass. The species involved was the European Robin, *Erithacus rubecula* (Turdidae), a small migratory bird [1]. Meanwhile, a magnetic compass has been demonstrated in more than 20 other bird species, mostly passerine songbirds [2], but also in homing pigeons *Columba livia domestica* [3,4], sanderlings, *Calidris alba* (Scolopacidae), a shorebird species [5], and in domestic chicken, *Gallus gallus* [6]. It was also shown in a number of other animals, involving members of all vertebrate classes, insects, crustaceans and mollusks [7]. Birds are still the best-studied group where magnetic orientation is concerned, and they are the only group where the principles of magnetoreception have begun to be understood. Much less is known about the function of the compass and the potential reception mechanisms in other animals; yet the little that is already known indicates that magneto-reception is not a uniform phenomenon, not even among vertebrates (e.g., [8]; see [7,9,10]). The findings suggest that the avian magnetic compass may be a unique development of birds, different from the mechanisms used by other animals.

Here, we will briefly review our present knowledge on how birds detect the direction of the geomagnetic field.

## 2. Demonstrating Magnetic Compass Orientation in Birds

**Figure 1:**
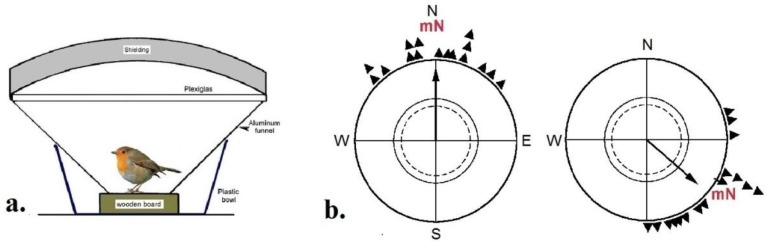
(**a**) Section through a frequently used test cage for recording the activity of a migratory bird [11]. (**b**) Orientation of robins during spring migration (**left**) in the local geomagnetic field and (**right**) with magnetic North turned by 120° to ESE (data from [13]). The triangles at the periphery of the circle mark the mean headings of individual birds, the arrow represents the grand mean vector based on these headings drawn proportional to the radius of the circle. The two inner circles indicate the 5% (dotted) and the 1% significance border of the Rayleigh test [14].

To demonstrate magnetic compass orientation in animals, one needs a behavior where the animal reliably prefers a direction. Here, the migratory behavior of birds proved most helpful: during the migration season, migratory birds undertake extended flights in their migratory direction. The urge to head in this direction is so strong that even captive migrants move in this direction in suitable circular cages (Figure 1a): they hop and flutter into the direction in which their free-flying conspecifics migrate. The distribution of their activity can be recorded [11] and from this, their heading calculated. Usually, a group of 10 to 15 birds in tested singly several times, and from the mean headings of these birds, a grand mean is calculated.—All behavioral data from migratory birds reported here were obtained this way.

With non-migratory species, the problem is to induce directionally oriented behavior. In pigeons, their urge to home after displacement produces reliable directional preferences (e.g., [3,4]); other non-migrants, like domestic chickens or zebra finches, *Taeniopygia guttata* (Estrildidae), were trained to prefer specific directions in conditioning experiments [6,12].

To test whether birds use the magnetic field as an orienting cue, they are tested in the local geomagnetic field and in a field where magnetic North is shifted by a certain angle with the help of a coil system: a corresponding shift in their directional preference shows that they indeed used the magnetic field as a compass (Figure 1b).

With respect to magnetoreception, observing oriented behavior means that the birds can derive meaningful directional information from the magnetic field in the given situation—their magnetoreception mechanisms are unimpaired.

## 3. Characteristics of the Avian Magnetic Compass

The same behavioral method was used to further analyze the functional properties of the magnetic compass of birds. The respective tests were performed at Frankfurt am Main, in a local geomagnetic field of 46 μT (microTesla) and 66° inclination, with European robins as test birds. They produced some surprising results, and it soon became evident that the avian magnetic compass is fundamentally different from the movable magnetized compass needles in the technical compass we humans use to orient ourselves.

### 3.1. The Functional Window

**Figure 2:**
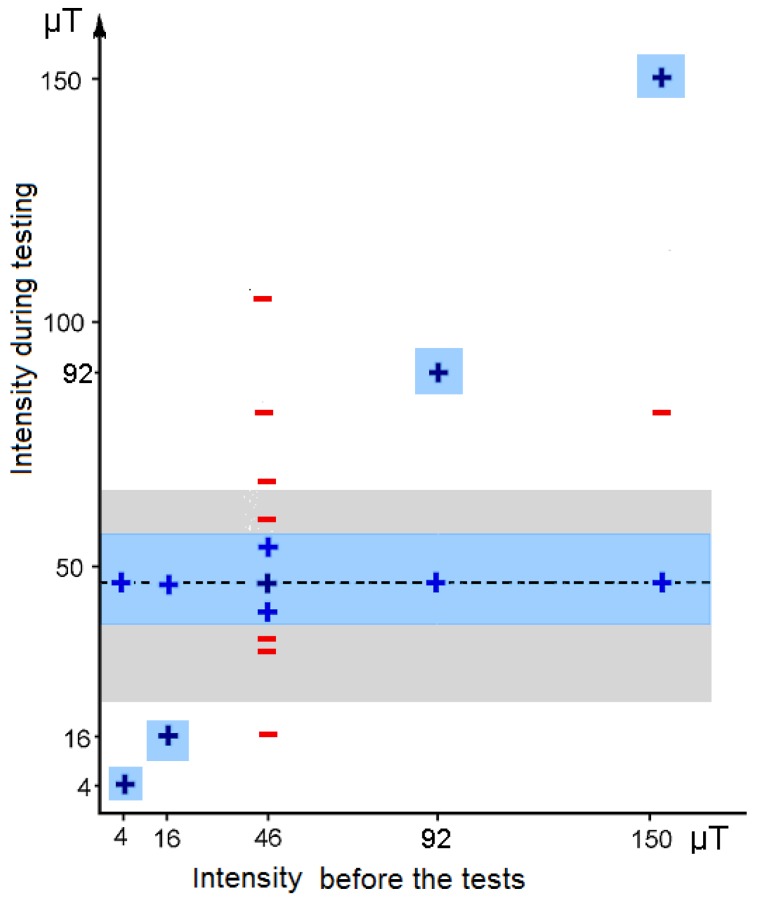
The functional window of the magnetic compass and its flexibility: orientation of robins in various magnetic intensities. Blue: +, oriented behavior; red: −, disoriented behavior. The dashed line marks the local intensity of the capture site, 46 μT. The blue zones indicate the estimated functional range of the magnetic compass in birds kept in the intensity indicated at the abscissa; the grey zone marks the intensity range presently found on Earth (data from [15,17,18]).

The magnetic compass of birds normally works only in a rather narrow intensity range around the intensity of the local geomagnetic field. Increasing or reducing the magnetic intensity by about 25–30% led to disorientation (Figure 2)—obviously, the birds could not read the magnetic field any more [15]. A similar functional window was also indicated in young domestic chickens [16]. At lower intensities, this is not so surprising, as it could mean that the intensity got below threshold, yet the disorientation at higher intensities seemed rather odd.

The functional window is not fixed, however, but proved rather flexible. Staying in an intensity outside the functional range induced the ability to orient in that intensity: e.g., being kept for 1 h in an intensity of 92 μT enabled birds to orient at this intensity that is twice the local intensity [17]. Adjustment to very low intensities took longer, but eventually, after staying a total of 17 h in this field, birds were able to orient at 4 μT, less than 1/10 of the local geomagnetic field [18].

Birds can thus orient in the intensity in which they are kept prior to the tests and, at the same time, they continue to be oriented in 46 μT, the local intensity of the capture and housing site. This shows that their ability to orient in other intensities does not involve a shift in the functional window. It does not represent an enlargement of the functional window either, as e.g., birds caught at 46 μT and then housed at 150 μT were able to orient at these two intensities, but not at the intermediate intensity of 81 μT (see Figure 2) [15]. Obviously birds have to experience new intensities directly to be able to derive magnetic directional information. The fast adjustment to intensities outside the normal functional window suggests neural processes altering the interpretation of the input rather than changes at the receptor level.

### 3.2. The Inclination Compass

**Figure 3:**
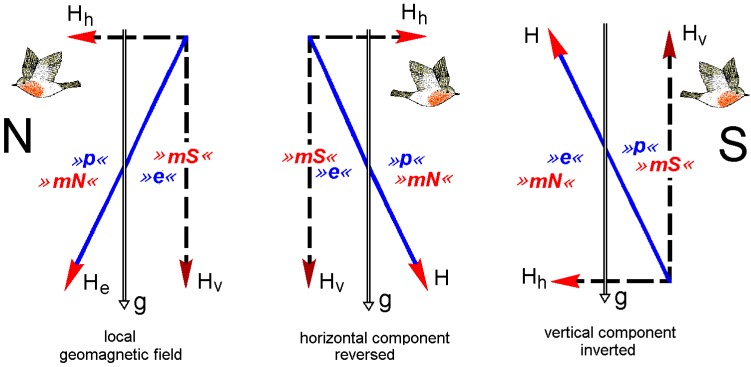
The avian inclination compass: cross-section through the magnetic field as seen from the West. N, S, geographic North and South; H, magnetic vector; He, vector of the local geomagnetic field; Hh, Hv. horizontal and vertical component of the magnetic field, with the red arrow tips indicating the polarity; the axial course of the field lines is indicated in blue. g, gravity vector indicating downward. Red »mN«, »mS«, *magnetic North* and *South*, the readings of a polarity compass; blue »p«, »e«, *poleward* and *equatorward*, the readings of the avian inclination compass. The robins’ flying direction indicates where the birds seek their spring migratory direction (after [19], modified).

In the northern hemisphere, the vertical component of the geomagnetic field points downward. When the vertical component was inverted so that it points upward, simulating a field of the southern hemisphere, robins reversed their directional preferences, that is, in spring, they now headed southward instead of northward [19]. In this test field (Figure 3c), the polarity is still pointing northward, and our technical compass would not indicate a difference. Robins, however, obviously ignore the polarity of the magnetic field; for them, inverting the vertical component had the same effect as reversing the horizontal component. They orient according to the axial course of the field lines (see Figure 3), their compass being a so-called *inclination compass.* This means that they do not distinguish between “magnetic North” and “magnetic South”, which are the readings of a compass based on polarity, but instead between “poleward”, where the field lines are pointing downward, and “equatorward”, where they point upward.

Meanwhile, an inclination compass has been demonstrated in some more avian species, among them several migrants (for review, see [2]) and the homing pigeon [4]). It has been found in all birds tested for it so far and appears to be a general characteristic of the avian magnetic compass.

### 3.3. Wavelength Dependency of Magnetic Orientation

The magnetic compass of birds requires light. Very young, inexperienced pigeons base their navigation on route information: they record the net direction of the outward journey with their magnetic compass and reverse it to obtain the homeward course [20]; when displaced in total darkness, they could not do so and departed randomly [21]. Migratory birds, too, can no longer orient in their migratory direction in darkness [22,23].

**Figure 4:**
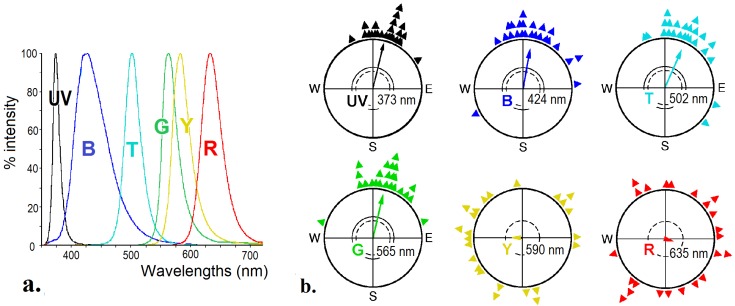
Orientation of robins under narrow band-lights of various wavelengths. (**a**) Spectra of the test lights produced by light-emitting diodes. (**b**) Orientation behavior under the various lights, with the peak wavelength indicated in the figure. The light intensity was about 8 × 10^15^ quanta/s·m^2^, except under UV, where it was only 0.8 × 10^15^ quanta/s·m^2^.—Symbols as in Figure 1b (data from [25,26,29,30]).

When birds were tested under various narrow-band lights, it became evident that the avian magnetic compass requires light from the short-wavelengths part of the spectrum. For these tests, the test cages were placed in a cylinder, the top of which was carrying light-emitting diodes (LEDs). Robins and other passerines tested under 373 nm UV, 424 nm blue, 502 nm turquoise, and 565 nm green light showed orientation in their migratory direction, but were disoriented under 590 nm yellow and 635 nm and 645 nm red light [24,25,26,27,28,29,30], see Figure 4. The same wavelength dependency is indicated in homing pigeons [31] and domestic chickens [14]; it also appears to be a characteristic common to all birds (for discussion, see [21]).

With 8 × 10^15^ quanta/s·m^2^, the light level under which magnetic orientation was observed is rather low. Under blue, turquoise and green light, it corresponds to the light about 45 min after sunset/before sunrise on a clear evening/morning at 50°N; under UV, it was even lower. Under brighter narrow-band lights, however, birds no longer prefer their migratory direction and show responses that are no longer controlled by their inclination compass [23,32].

## 4. Magnetoreception Based on Spin-Chemical Processes

The unusual characteristic of the avian magnetic compass—functional window, inclination compass and its dependency on short-wavelength light—would seem to rule out induction or mechanisms involving permanently magnetic material, because these respond to the polarity of the magnetic field, which birds evidently ignore. This implied an unusual mechanism of magnetoreception.

A first mechanism in agreement with the properties of the avian magnetic compass was proposed by Schulten [33,34] and 2000 described in detail by Ritz and colleagues [35]: their Radical Pair Model of Magnetoreception suggests a “chemical compass” based on spin-chemical processes in photopigments that interact with the geomagnetic field.

### 4.1. The Radical Pair Model

The model forwarded by Schulten and Ritz [35] proposes that absorption of a photon raises a receptor molecule into an excited state and leads to a light-activated electron transfer from a donor to an acceptor, thus generating a spin-correlated radical pair. By interconversion, singlet states radical pairs with an antiparallel spin are transformed into a triplet states with parallel spin and *vice versa*. The ratio singlet/triplet depends, among other factors, on the alignment of the receptor molecule in the external magnetic field and could thus mediate information on magnetic directions [33]. This brief account of the reaction scheme is greatly simplified; for details on the physical background, requirements and calculations, see e.g., [35,36,37,38,39,40].

**Figure 5:**
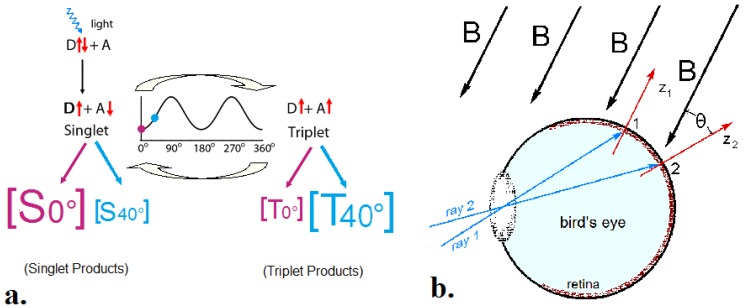
The Radical Pair Model of magnetoreception. (**a**) Scheme of the radical pair mechanisms proposed by Ritz and colleagues [35]. After photon absorption, a radical pair is generated by an electron transfer from a donor (D) to an acceptor (A), with the ratio singlet/triplet depending on the alignment of the molecule in the external magnetic field. The red arrows represent the spins of the electrons. The changing singlet/triplet ratio as a function of the alignment is indicated in the inner diagram; note that 0° = 180° and 90° = 270°. The amount of singlet and triplet products is symbolized for a parallel alignment and a 40° alignment. (**b**) Light rays are projected onto the retina, activating receptor cells that are aligned at different angles with respect to the direction of the magnetic vector B (from [33], modified).

To obtain directional information by a radical pair mechanism, birds must be able to compare the singlet or triplet yield in different spatial directions. Because of this, Ritz and colleagues [35] suggested the eye as the site of magnetoreception: light is available, and because of the near-spherical form of the eyeball, all spatial directions are represented. Assuming that the receptor molecules are arranged similarly in receptor cells all across the retina (Figure 5b), this would lead to different amounts of singlet and triplet products. Provided birds are able to sense and compare the different quantities of these products, this would result in a specific activation pattern across the retina, which is centrally symmetric to the field lines and thus could indicate magnetic directions [35].

The radical pair reaction does not depend on the polarity of the magnetic field, but only on the axial course of the field lines, with the response in an alignment parallel to the magnetic vector equal to that in an antiparallel alignment (see Figure 5a). Hence, it can necessarily give only information on the course of the field lines and thus provides an explanation for the functional mode of the avian inclination compass. The activation pattern on the retina also changes with intensity [35], so that the functional window and its flexibility are likewise explained: in a field with intensity outside the functional window, birds are confronted with a yet unfamiliar pattern, which is confusing at first. The pattern, however, is likely to retain its central symmetry with respect to the field lines, and hence birds would be able to interpret it after a while, thus, regaining their magnetic compass.

### 4.2. Testing the Model

The Radical Pair Model of magnetoreception makes several predictions that can be tested. The initial photon absorption would make magnetoreception light-dependent. This is indeed the case (see Figure 4): the avian magnetic compass requires short-wavelengths light in all bird species tested so far [25,26,27,28,29,30].

**Figure 6:**
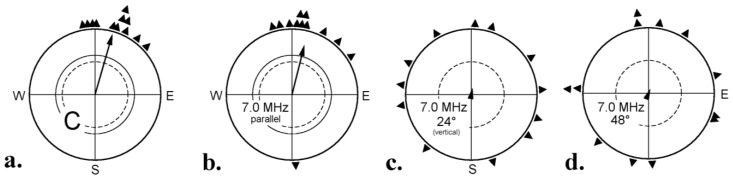
Testing robins with radio frequency fields of 7 MHz, 470 nT, added in different alignments with respect to the vector of the local geomagnetic field. (**a**) Control: geomagnetic field only; (**b**) radio frequency field added parallel to the magnetic vector, that is 24° to the downward direction; (**c**) added vertically, 24° to the magnetic vector; (**d**) added 48° to the magnetic vector, which means 24° to the downward direction—Symbols as in Figure 1b (data from [44]).

A diagnostic test for an involvement of radical pair processes is to apply radio frequency fields in the MHz (MegaHertz)-range, as they would interfere with the singlet-triplet interconversion [41,42]. Here, the alignment of the applied oscillating field with respect to the vector of the static background field is important [43]. In critical tests, the radio frequency fields were therefore added in different alignments to the local geomagnetic field with its inclination of 66°. A 1.315 MHz and a 7 MHz field of 470 nT, added parallel, did not disrupt orientation, but when the same fields were applied vertically, *i.e*., at an angle of 24° to the vector of the geomagnetic field, or at an angle of 48°, the birds were disoriented, indicating that they lacked meaningful directional information (Figure 6) [44,45]. These findings support the Radical Pair Model of magnetoreception.

The comparison of the two fields in Figure 6b,d is of special interest, because both were aligned 24° with respect to the downward direction. For the freely moving test birds, these alignments were identical, but in one case, it meant parallel to the vector of the geomagnetic field, where it did not disrupt orientation, in the other case, an angle of 48° with respect to this vector, where it had a disorienting effect. This excludes non-specific effects, as it clearly shows that not the radio frequency field *per se* was disrupting, but that its alignment with respect to the vector of geomagnetic field was crucial [44].

The experiments described above were performed under 565 nm green light. An interference of radio frequency fields with orientation of robins was also observed under 373 nm ultraviolet, 424 nm blue and 501 nm turquoise light [23]: the same radical pair mechanism is underlying the magnetic compass within this wavelengths range. Conditioned directional responses to the magnetic field in domestic chickens and zebra finches were also disrupted by radio frequency fields [16,46].

The tests mentioned above involved single frequencies. In a broad-band radio frequency field, including frequencies from 0.1 to 10 MHz of 85 nT, add vertically to the geomagnetic field, the birds were likewise disoriented [44]. Recently, it was reported that man-made electromagnetic noise in the frequency range from 0 to 5 MHz also caused disorientation, even when the fields were only 10 nT and below [47]. The basis of this extreme sensibility remains to be determined.

### 4.3. Further Analysis of the Radical Pair Mechanism

Doing “behavioral spectroscopy” by testing birds in radio frequency fields of various frequencies and intensities allowed a further analysis of the radical pair processes involved in the avian magnetic compass. The tests described below used oscillating fields added vertically, that is, at an angle of 24° with respect to the geomagnetic vector.

**Figure 7:**
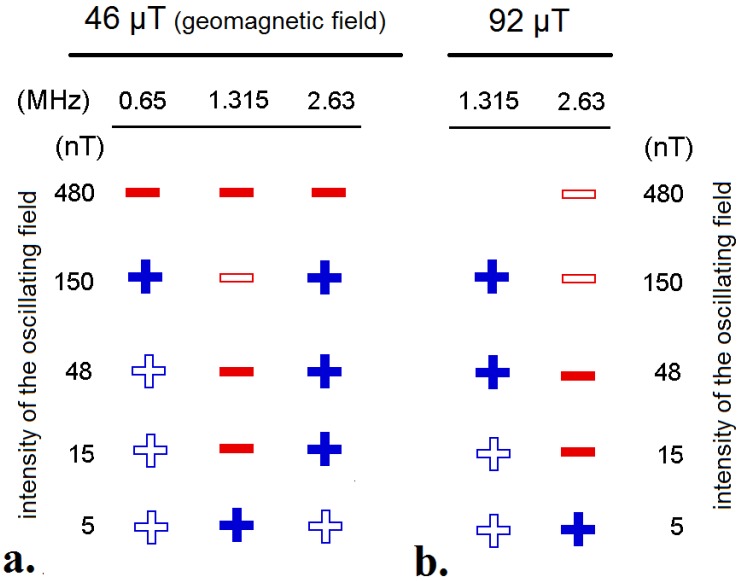
Summary of the tests with different frequencies and different intensities: (**a**) in the geomagnetic field of 46 μT and (**b**) in a 92 μT field, twice that intensity. Red: −, disoriented behavior, indicating an interference with magnetoreception; blue: + no disruptive effect of the respective oscillating field. Solid symbols: results from experiments, open symbols: inferred from the other results under the assumption of monotony (based on data from [48]).

In oscillating fields with an intensity of 480 nT, frequencies of 0.01 and 0.03 MHz did not disrupt the birds’ orientation. In a 0.1 and a 0.5 MHz field, axial behavior was observed, with birds preferring their migratory direction and the opposite direction [48]; such behavior is often observed in situations where the magnetic compass is at the edge of its range of operation [28]. At frequencies of 0.65 MHz and higher, the birds were no longer oriented (see Figure 7a), indicating a disruptive effect of these radio frequencies on magnetoreception [48].

Fields oscillating with frequencies whose periods are longer than the lifetime of the radical pair are effectively static. It appears reasonable to assume that the onset of the effect of oscillating fields concurs with the transition to fields with sufficiently high frequencies to oscillate during the coherence lifetime of the radical pair. Hence, one can estimate the coherence time of the radical pair as the reciprocal of the threshold frequencies. The series of findings reported above suggest a coherence time of about 2–10 μs [48].

Another series of experiments was devoted to the sensitivity of the response at different frequencies, focusing on the Larmor frequency of the electron, which, in the in local geomagnetic field, was 1.315 MHz. A field of half the Larmor frequency, 0.65 MHz, and twice the Larmor frequency, 2.63 MHz, had a disruptive effect when presented with an intensity of 480 nT, but did no longer interfere with magnetoreception when the intensity was decreased to 150 nT or below (Figure 7a). A field of the Larmor frequency of 1.315 MHz, in contrast, disrupted orientation even when it was as weak as 15 nT (Figure 7a) [48]. Doubling the static background field increases the Larmor frequency to 2.63 MHz. The respective tests in a static 92 μT field revealed that the highly sensitive response indeed shifted to 2.63 MHz, while the frequency of 1.315 MHz lost its disruptive effect at 150 nT and 48 nT (Figure 7b) [48].

This very sensitive response at the Larmor frequency suggested specific properties of the radical pair underlying magnetoreception. Such a strong resonance is expected only for a radical pair in which one of the radicals is devoid of atoms, such as hydrogen and nitrogen whose nuclei have magnetic moments. This special radical contains an electron spin that has no magnetic interactions other than with the external magnetic field [48]. It could, thus, act as a “probe” in a reference-probe system, increasing the overall sensitivity to the magnetic field considerably [49,50]. Hence it would be particularly suitable as a magnetic sensor—it appears to have the optimal design for detecting magnetic directions (for theoretical considerations and details, see [48,49,50]).

The responses of the birds in the experiments with applied radio frequency fields are thus in agreement with the Radical Pair Model of magnetoreception [35], indicating that the avian magnetic compass is probably based on radical pair processes.

## 5. The Receptor Molecule

When Ritz and colleagues [35] proposed the Radical Pair Model, they suggested cryptochrome as receptor molecule, because in these molecules, photon absorption leads to the formation of radical pairs [51]. Cryptochrome, a blue-light photoreceptor with flavin as chromophore, was first described in plants [52], where it is involved, e.g., in the control of hypocotyl growth, photoperiodic induction of flowering and other circadian and photoperiodic responses (for review, see [53,54]). Cryptochromes were also found in a number of animals, where they are involved in circadian rhythms and their entrainment (see [53,54,55]); in 2002, they were first reported in birds [56,57,58]. Meanwhile, four types of cryptochromes have been identified in the eyes of chickens [56,57,58,59,60] and passerines [59,61,62,63,64]: cryptochrome 1 in two splice products, Cry1a and Cry1b [61], cryptochrome 2 and cryptochrome 4.

### 5.1. Localization of Cryptochrome 1a

Most of the studies on cryptochromes in the avian eyes concern mRNA, and the exact location of the protein remains unclear. In a few cases, however, antibodies were used to mark cryptochrome *in situ*. A cryptochrome 1, probably Cry1b [62], was reported from the nuclear layer and the displaced ganglion cells of a garden warbler, *Sylvia borin*, showing migratory activity; it was discussed as being involved in magnetoreception. However, it was not found in zebra finches [62], although a magnetic compass based on radical pair processes is also indicated in this species [46]. Cryptochrome 4 was also found in ganglion cells and, in a smaller amount, in the inner nuclear layer and the photoreceptor cells of chickens [60].

**Figure 8:**
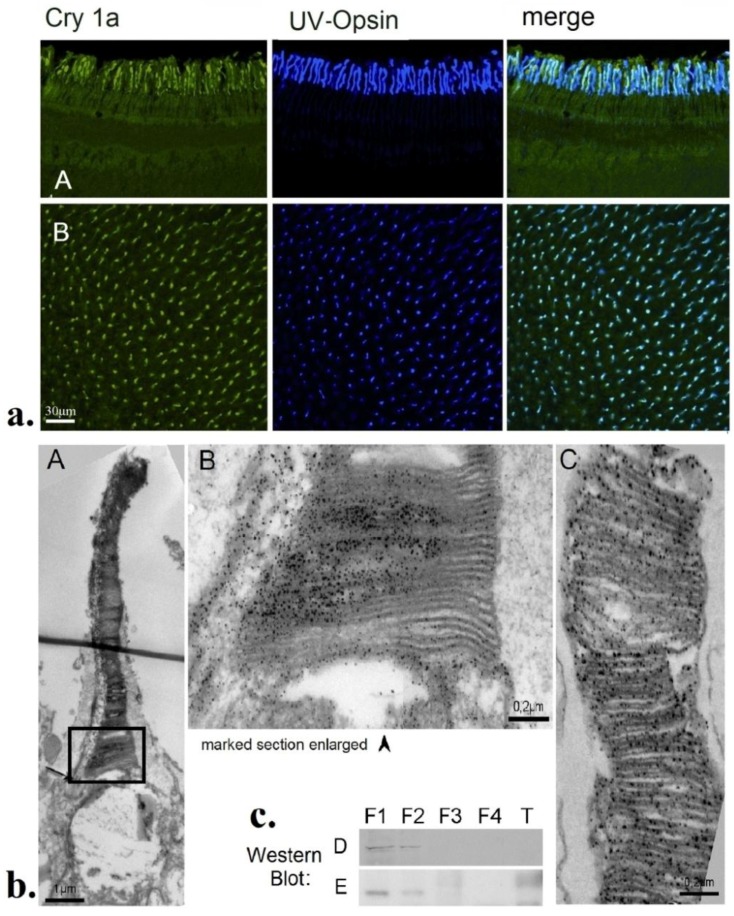
Localization of cryptochrome 1a (Cry1a) in the retina of robins and chickens. (**a**) Immuno-labeling of Cry1a and UV-opsin and their co-localization in the retina of robins. A, Vertical section through the outer part of retina; B, whole mount of a retina. (**b**) Electron-microscopic images of the outer segments of the UV/V-cones, with labeled Cry1a visible as dark dots along the disk membranes. A, entire outer segment of a chicken V-cone. B, higher magnification of the lower part of this outer segment. C, Part of the outer segment of an UV-cone of a robin. (**c**) Western blots of robin (D) and chicken (E) retina showing Cry1a in the cytosol and membrane fraction. F1, cytosolic fraction; F2, membrane fraction; F3, nuclear fraction; F4, cytoskeletal fraction; T, tongue tissue as control (from [59]).

The most promising candidate so far is cryptochrome 1a. Using a specific antiserum, Nießner and colleagues [59] found marked Cry1a in a particular type of photoreceptor cells, which was identified by an antiserum against SWS1-opsin as the V-cones of chickens and the UV-cones of robins (Figure 8a). Western blots (Figure 8c) showed Cry1a in the cytosolic and the membrane fraction. In electron-microscopic images, it was found located at the membranes of the disks in the outer segments of both species (Figure 8b). Double labeling indicated that all UV/V cones contain Cry1a, and that Cry1a is in no other cones. Differences between chickens and robins were not observed [59].

The UV/V cones of birds have thus been identified as probable receptor cells for magnetic directions. These cones represent the least frequent cone population, with on average only 9% of the cones belonging to this type [65,66]. As the magnetic field-induced activation pattern has smooth and gradual transitions, a low-density detector system is sufficient to detect these signals [67]. Also, the other cones of birds contain colored oil droplets, which act as selective cut-off filters; only the UV/V cones have transparent oil droplets that transmit all wavelengths (see e.g., [68,69]), also the short wavelengths absorbed by cryptochrome. So this could also be the reason for their additional function as magnetoreceptors [67].

With respect to the requirements of the Radical Pair Model, the location of Cry1a in the UV/V cones appears well-suited to perceive magnetic directions: Cry1a seems to be attached to the membranes of the disks, so that the reactions of the various Cry1a molecules do not cancel each other, but can add up to a joint response of the receptor cell [38]. However, in this respect, the mechanism appears to be rather robust: calculations indicated that a certain amount of static disorder of the receptor molecules would be permitted without disrupting the function as magnetoreceptors [38,70,71,72]. The UV/V cones are distributed more or less evenly across the retina in robins as well as in chickens (see Figure 8a(B) [59]) so that all spatial directions are represented. This would lead to the activation pattern across the retina proposed by Ritz and colleagues [35] to provide birds with directional information.

How the radical pair processes in cryptochrome give rise to this pattern is still the subject of speculations. Several transduction mechanisms have been suggested: long-lived electric dipole moments [73]; spin entanglement and spin orbit coupling [74,75] have been considered; the amount of singlet or triplet products might affect membrane channels directly or indirectly by some binding partner (see [76]). However, since there are no indications for two separate outputs in the UV/V cones, the radical pair mechanism could interact with the signaling cascade of the SWS1-opsin to affect the state of the channels in the outer membrane [59].

### 5.2. Light-Activation of Cryptochrome 1a

In their antiserum study, Nießner and colleagues [59] found labeled Cry1a only at the disks in the outer segment of the UV/V cones, but not in the inner segment where it is produced. This suggested the intriguing possibility that the antiserum, which was raised against a specific sequence at the C-terminal domain, marked only activated Cry1a. A critical test confirmed this: after exposure to 30 min of darkness, no Cry1a was found labeled. When the 30 min period of darkness was followed by 5 min of UV light, however, a considerable amount of marked Cry1a was visible [77]. This fast response to light excludes a degradation of the protein in the dark and its later reconstitution, because 5 min are too short to synthetize Cry1a and transport it to the outer segment. Instead, it suggests light-activation leading to a conformational change: in the dark, the epitope of the antiserum appears to be inaccessibly hidden inside the complex molecule; light leads to the exposure of the C-terminus and thus allows the antiserum to bind [77].

**Figure 9:**
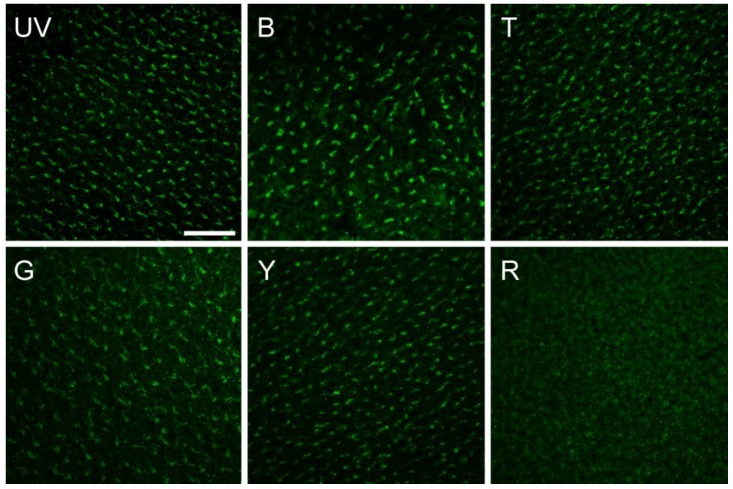
The amount of activated Cry1a, labeled with a specific antiserum, in the retina of chickens after illumination with light of various wavelengths. UV, 373 nm UV light; B, 424 nm blue light; T, 502 nm turquoise light; G, 565 nm green light; Y, 590 nm yellow light; R, 635 nm red light, see Figure 4a (from [77]).

The antiserum thus offered the opportunity to analyze the light activation of Cry1a under specific wavelengths *in vivo*. In the respective study, chickens were exposed for 30 min to the same lights that had been used for the behavioral tests with robins (see Figure 4a), and then the activation status of Cry1a was checked. The results are given in Figure 9: illumination with ultraviolet (UV-A), blue and turquoise light produced activated, labeled Cry1a; after illumination with green and yellow light, the labeled amount of Cry1a was somewhat smaller, and no labeling was found in red light [77].

A comparison with the behavioral data in Figure 4b shows that Cry1a is labeled under all light conditions where robins show oriented behavior—the activation of Cry1a concurs with detecting magnetic directions; it appears to be a necessary condition. This supports the role of Cry1a as receptor molecule for magnetic compass information.

Only under yellow light, the situation is puzzling, as there is a certain amount of Cry1a labeled, but no orientation is observed in birds ([26], see Figure 4b). The reasons are unclear. Interferences of yellow light with orientation based on the radical pair mechanisms have also been observed in other test situations (e.g., [74]), but the disruptive effect of yellow light is not yet understood (see [23] for discussion).

### 5.3. The Flavin Cycle and the Radical Pairs

**Figure 10:**
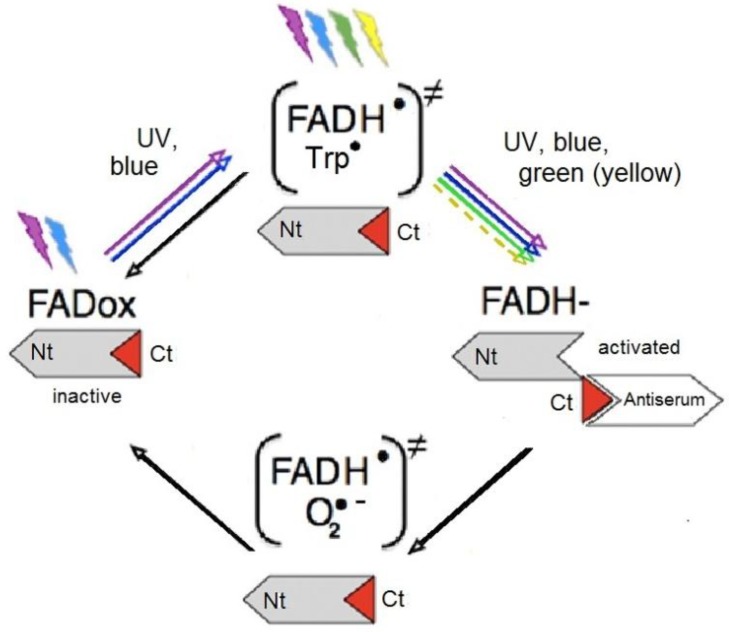
The redox cycle of flavin. FADox, oxidized flavin; FADH^●^, photo-reduced neutral radical form; FADH^−^, fully reduced form. Nt, nitrogen-terminus; Ct, carboxy-terminus of the Cry1a, with the antiserum-binding epitope in red. In parentheses, radical pairs, black arrows indicate light-independent reactions (from [77] after [79], modified).

A comparison of the observed light activation of Cry1a (Figure 9) with the known absorption curves of most cryptochromes gives some indications on the nature of the activated form that is marked by the antiserum. Flavin undergoes a redox-cycle [79]: the oxidized form, FADox, absorbs UV and blue light up to about 500 nm to be photo-reduced to the semiquinone, which, in robins, is the neural semiquinone FADH^●^ (Ahmad, pers. comm). It forms a first radical pair FADH^●^/Trp^●^ with tryptophan (Figure 10). FADH^●^ can be re-oxidized directly in a light-independent reaction, or, if light is present, can absorb UV, blue, and green light up to about 570 nm to be further reduced to the fully reduced form, FADH^─^. This fully reduced form of flavin is re-oxidized in a light-independent reaction, generating a second radical pair, possibly FADH^●^/O2^●─^ (see Figure 10) [79].

Under UV, blue and turquoise light, the full cycle will run, with all forms of flavin generated and present at the same time in a dynamic equilibrium [79]. Most interesting is the situation under green light: here, the first step, the photoreduction of FADox to the semiquinone FADH^●^ cannot take place, and the first radical pair, FADH^●^/Trp^●^, is not generated. However, before the exposure to green light, the chickens had been kept in daylight, so that a certain amount of FADH^●^ can be assumed to have been present at the beginning of the exposure. This semiquinone can be further reduced by green light to the fully reduced form FADH^─^, and this, in turn, can be re-oxidized independently of light, forming the second radical pair. That is, as long as there is a supply of FADH^●^ left, the second part of the cycle can still run. The same applies to the robins tested under 565 nm green light in the behavioral experiments: these birds had been kept in “white” light before. Labeled, that is activated, Cry1a was observed after illumination with light that prevents the first step of photo-reduction, but only under green, not under red light. This points out the crucial role of the step to the fully reduced form FADH^─^, which seems to be where the conformational change takes place (see Figure 10) [77]. This activation of Cry1a and the observed orientation under green light in birds that had been exposed to “white” light before (see Figure 4b [25]) suggests that not the first radical pair FADH^●^/Trp^●^ generated during photoreduction is the crucial one for magnetoreception, but the second one formed during re-oxidation [77].

This reaction suggested here for avian Cry1a is unusual insofar, as in most cryptochromes analyzed, the conformational change occurs during the first step of photoreduction, namely when the radical FADH^●^/Trp^●^ is generated, which in most cases is considered to be the signaling form [40,80,81,82]. It should be considered, however, that the role of Cry1a as receptor molecule for magnetoreception is different from what cryptochromes normally do, namely signaling the presence or absence and the amount of light—in the avian magnetic compass, cryptochrome has to indicate directions derived from the different singlet/triplet ratio [35]. The fully reduced form FADH^─^ appears to be the signaling one [77], and it could be rendered magnetically sensitive, because the ratio singlet/triplet, which depends on the alignment in the receptor molecule in the geomagnetic field, could affect the efficiency of re-oxidation (see [77] for a more detailed discussion).

The radical pair FADH^●^/O2^●─^ formed during re-oxidation would fulfill the condition of one radical being devoid of hyperfine interactions and thus being particularly suited to detect magnetic directions [48,49,50,83]. Yet theoretical consideration seem to indicate that O2^●─^ itself might be problematic because of fast spin relaxation, possibly too fast for being affected by the alignment with the magnetic field [50,84], but any other radical with the required characteristics could take its place (see [50] for a detailed discussion).

In summary, for signaling magnetic directions, another type of radical/radical pair could be more suitable than the one normally signaling light in cryptochromes. If this were so, it would not be surprising if evolution had shaped the mechanism and adapted it specifically to the required task.

## 6. Processing Magnetic Directional Information

The transduction and processing of magnetic compass information is still poorly known, with many questions still open. 

One of the problems arises from magnetic information being sensed together with visual information in the UV/V receptors of birds: these receptors contain two types of photopigments, namely the UV or violet sensitive SWS1-opsin which is affected by light, but not by the magnetic field, and additionally the cryptochrome which absorbs blue light [54] and is modulated by its changing alignment with respect to the direction of the geomagnetic field [35]. Thus the level of activation of the UV/V cones depends on the incident light falling on the UV-opsin as well as on the activation of Cry1a. Behavioral data indicate that the reception of magnetic directions does not dependent on the activation of the UV/V cones by light—it occurs under UV light that activates the UV cones as well as under narrow band green light that is not absorbed by SWS1-opsin ([30], see Figure 4b). Hence, at the reception level, magnetoreception and vision appear to be largely independent from each other, yet the output of the UV/V cones represents visual as well as magnetic information. Since the UV cones are fully integrated in the tetra-chromatic color system of birds, where ultraviolet vision plays an important role in social contexts like mate choice or for recognizing food like ripe fruits [85], visual information and the magnetic information must be separated. How and where this separation occurs is still unclear; several possibilities have been discussed in [67].

The processing of magnetic compass information in the brain, and where it takes place, is likewise not yet well known. Early electrophysiological studies [86,87] indicated a central role of the visual systems: responses to changes in the direction of the ambient magnetic field were observed in the nBOR, a part of the accessory optic system, and in the *stratum griseum et fibrosum superficiale* of the *tectum opticum* of pigeons, but only in the presence of light. Individual units responded with a distinct increase in spike frequency in a particular alignment of the magnetic field, which varied between cells. Processed together, they would represent all directions and could add up to a spatial pattern representing magnetic directions [86,87].

Later histological studies, involving markers for neural activity and neuronal tracings, confirmed the important role of the visual system, in particular the thalamofugal pathway [88]. In garden warblers showing nocturnal migratory activity, increased activity was observed in a specialized part of the visual Wulst, cluster N [89], which was interpreted as an area processing magnetic compass information. This seemed to be confirmed by a behavioral study: birds with cluster N lesioned could orient by celestial cues, but no longer by the geomagnetic field [90]. However, no increased activity in Cluster N was observed in a day migrant [91]. This leaves the exact role of cluster N unclear: either processing of magnetic compass orientation during day and night involves different parts of the brain [91], which does not seem very likely, or cluster N controls aspects of conditions that are essential for nocturnal orientation by the magnetic field, but is not directly involved in the processing magnetic directional information itself. 

Electrophysiological responses to changes in the direction of the magnetic field were also reported from the hippocampus of pigeons [92], a major center representing spatial information. In the brain of zebra finches, too, a directionally changing magnetic field caused some activation, which was most pronounced in the hippocampal subdivision [93]. In behavioral tests, however, birds whose hippocampus was lesioned were able to orient with their magnetic compass [92]. This appears to suggests that the hippocampus may not be involved in the direct processing of magnetic information that makes the magnetic compass available to birds, but rather in using the magnetic compass e.g., for integrating it with landmarks and other navigational factors to establish a directionally oriented map-like representations of the lay of the land. 

Altogether, the first studies analyzing the transmission and processing of magnetic compass information indicate an involvement of the visual system, but many more studies will be necessary to clarify where exactly and how this information is processed in the brain. 
